# Heyde Syndrome Complicated by a Dieulafoy Lesion

**DOI:** 10.31486/toj.19.0072

**Published:** 2020

**Authors:** Abdullah Noor, Dustin Abadco

**Affiliations:** ^1^ The University of Queensland Faculty of Medicine, Ochsner Clinical School, New Orleans, LA; ^2^ Department of Hospital Medicine, Ochsner Clinic Foundation, Kenner, LA

**Keywords:** *Angiodysplasia*, *Dieulafoy lesion*, *gastrointestinal hemorrhage*, *Heyde syndrome*

## Abstract

**Background:** Heyde syndrome, a triad of aortic stenosis, von Willebrand factor deficiency, and gastrointestinal (GI) bleeding from angiodysplasia, is a disease of the elderly. A Dieulafoy lesion, a specific type of angiodysplasia, is a large, tortuous, submucosal end artery that penetrates through the gastric mucosa and can cause life-threatening GI bleeding. We present a case of Heyde syndrome complicated by a Dieulafoy lesion.

**Case Report:** A 72-year-old female presented with GI bleeding evidenced by black tarry stool for 7 days. Hemoglobin (Hgb) level was as low as 6.0 g/dL. Double-balloon enteroscopy (DBE) revealed 2 jejunal angiodysplasias that were treated with argon plasma coagulation. The patient continued to have dark stools after discharge. Repeat complete blood count showed Hgb of 6.2 g/dL, and repeat DBE showed a 1-mm focus of active bleeding in the proximal jejunum consistent with a Dieulafoy lesion. The lesion was successfully treated with argon plasma at 1 L/min and 25 watts. At follow-up 1 year later, the patient had had no GI bleeding symptoms since discharge.

**Conclusion:** This case adds evidence that a Dieulafoy lesion is a potential complication of Heyde syndrome. Dieulafoy lesions can be life-threatening, so documenting occurrences that are complications of Heyde syndrome is important because of the potential for an increasing incidence of Heyde syndrome in the aging population.

## INTRODUCTION

Heyde syndrome, a triad of aortic stenosis (AS), von Willebrand factor (VWF) deficiency, and gastrointestinal (GI) bleeding from angiodysplasia,^1^ was first described by Edward C. Heyde in a case series of 10 patients who concomitantly had AS and gastric angiodysplasia.^2^ AS is common among the elderly,^1^ and its prevalence increases with age, from 1% to 2% at age 75 years to 6% at age 85 years.^3^ Heyde syndrome is reported in approximately 25% of patients with severe AS.^1^ The association between AS and GI bleeding has been questioned, but evidence of GI bleeding resolution after surgical aortic valve replacement reinforced the association.^1^ Hospitalizations and mortality among patients with Heyde syndrome have been increasing. From 2007 to 2014, data acquired from the National Inpatient Sample showed that hospitalizations and all-cause inpatient mortality associated with Heyde syndrome increased 29.16% (from 48 to 62 per 100,000) and 22.70% (from 3.7 to 4.54 per 100,000), respectively.^3^

A Dieulafoy lesion, a rare and specific type of angiodysplasia, is a large, tortuous, submucosal end artery that penetrates through the gastric mucosa over time.^4^ The lesion can eventually perforate and cause severe GI bleeding.^4^ French surgeon M. T. Gallard first identified the lesion in 1884 while attempting to describe 3 cases of massive GI bleeding, and Paul Georges Dieulafoy provided details about the lesion in 1898.^5,6^ The diameter of the artery in a Dieulafoy lesion can be 1 to 3 mm at the muscularis mucosae level, much larger than the diameter of normal arteries at that level.^4,7,8^ Common locations for a Dieulafoy lesion are the upper stomach, distal stomach, duodenum, and large bowel.^4,9^ Because a Dieulafoy lesion tends to protrude through the mucosa, a tiny erosion of the mucosa overlying the artery can result in massive bleeding, leading to a poor prognosis that can include death.^8,9^ The mortality rate for patients with a Dieulafoy lesion can be as high as 80%.^4^ Dieulafoy lesions account for approximately 1% to 2% of acute GI bleeds but are commonly underrecognized.^4^

We present a case of Heyde syndrome complicated by a Dieulafoy lesion in an elderly female and discuss the processes that may contribute to the formation of this type of lesion.

## CASE REPORT

A 72-year-old female with a medical history of AS, diabetes mellitus type 2, chronic kidney disease stage 3, gout, colon cancer treated with resection and colostomy bag placement 18 years prior, hyperlipidemia, hypertension, and morbid obesity presented with GI bleeding evidenced by black tarry stool in her colostomy bag for 7 days. Associated symptoms included fatigue, nausea, and decreased appetite. The patient reported no vomiting, abdominal distension, or abdominal pain. She had not recently taken any nonsteroidal antiinflammatory drugs. Echocardiography 1 month prior to presentation showed aortic valve area of 1.24 cm^2^, aortic valve mean gradient of 39 mmHg, and aortic orifice peak velocity of 4.11 m/s, indicative of moderate to severe AS.

The patient was initially seen at a regional hospital where her hemoglobin (Hgb) level was as low as 6.0 g/dL. During her stay at the regional hospital, she underwent push enteroscopy, colonoscopy, tagged red blood cell (RBC) scan, and angiography. The RBC scan showed delayed images for tracer accumulation in the right colon/terminal ileum. She was transfused 2 units of packed RBCs on day 4 of hospitalization and 3 units of packed RBCs on day 6. She was transferred to our tertiary care center on day 7 of hospitalization.

On arrival at our hospital, the patient's vital signs were within normal limits. She was alert and oriented. Her colostomy bag was located on the right lower quadrant of the abdomen and contained black tarry stool. Erythema was visible around the colostomy site. She had an existing colectomy midline wound with a linear bandage beginning below the xiphoid process and crossing the umbilicus. Other significant abdominal findings included abdominal distension and tenderness at the periumbilical and hypogastric region on deep palpation. A large hernia protruded from the pelvic region. Cardiovascular examination was significant for systolic ejection murmur, III/VI in intensity on the Levine scale, in the aortopulmonary area. Eye examination revealed conjunctival pallor.

On admission, the patient's laboratory results were significant for Hgb of 8.3 g/dL and a platelet count of 96 platelets/μL. Video capsule endoscopy showed blood in her proximal small bowel, and double-balloon enteroscopy (DBE) showed jejunal angiodysplasia that was treated with argon plasma coagulation. Repeat DBE on day 3 of hospitalization at our facility showed another small jejunal angiodysplasia that was also treated with argon plasma coagulation. Her Hgb remained stable during admission. She was discharged after 3 days of hospitalization with home health to resume wound care for her abdominal wound.

The patient continued to have dark stools after discharge. She was readmitted to the regional hospital 10 days after discharge. Repeat complete blood count showed Hgb of 6.2 g/dL. She was transferred back to our hospital the next day after being transfused another 2 units of packed RBCs. Repeat DBE showed a 1-mm focus of active bleeding in the proximal jejunum consistent with a Dieulafoy lesion (Figure).

**Figure.**
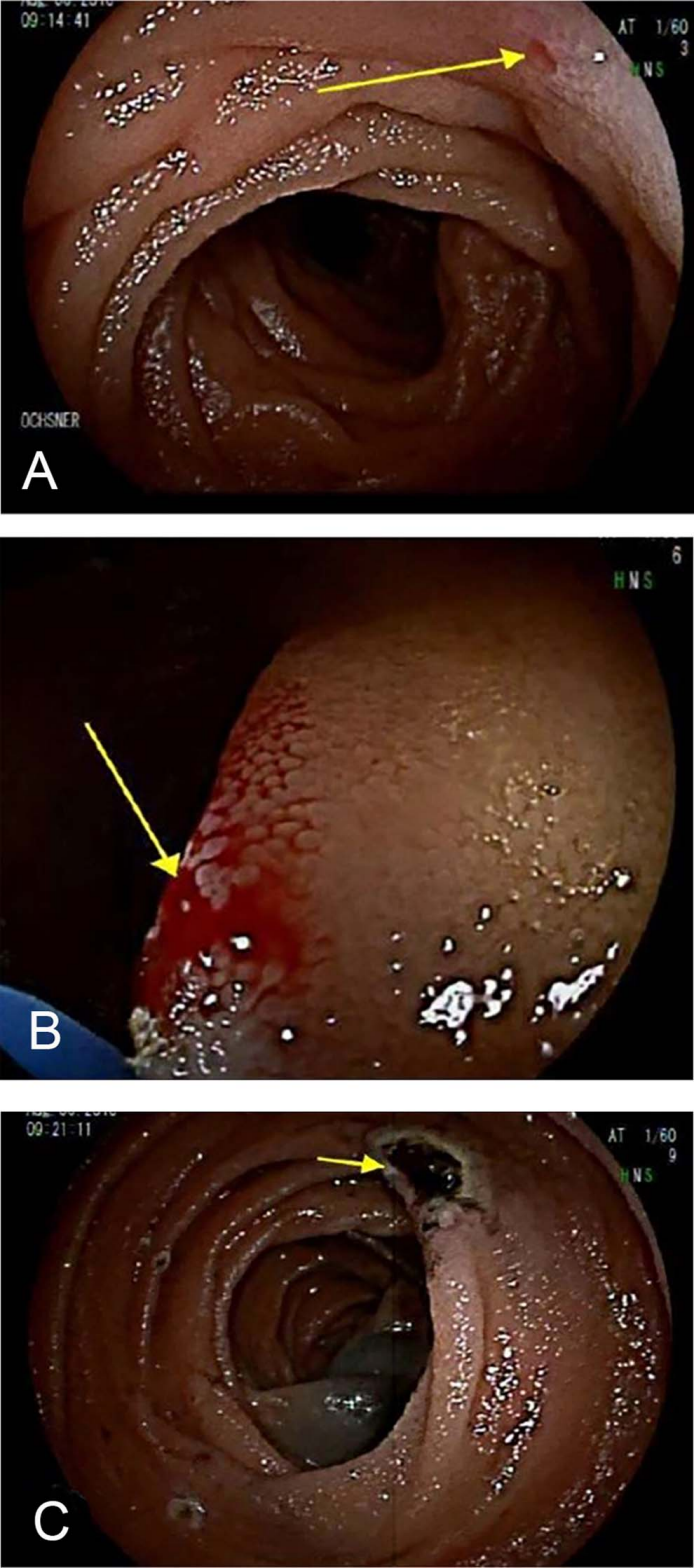
**(A) Dieulafoy lesion at the proximal jejunum. (B) Bleeding from the lesion. (C) Lesion after treatment with argon plasma at 1 L/min and 25 watts.** (Photos by Daniel Raines, MD).

The lesion was treated with argon plasma at 1 L/min and 25 watts. Two homeostatic clips were placed to prevent further bleeding. India ink 0.3 mL was injected to tattoo the area. The patient's Hgb remained stable at 7.9 g/dL during the postoperative observation period. After 2 days, she was discharged home. At follow-up 1 year later, she reported no GI bleeding symptoms since discharge.

## DISCUSSION

Our patient had findings characteristic of Heyde syndrome: history of AS, persistently low Hgb, low platelets, and angiodysplasia (the Dieulafoy lesion).

Our patient's VWF levels were not checked, but VWF deficiency can lead to low platelets. VWF, a high molecular weight multimer, is necessary in maintaining normal coagulation homeostasis, and its role in angiogenesis has been described as well.^10^ With regard to its role in homeostasis, VWF recruits platelets and initiates platelet aggregation by activating the glycoprotein Ib-IX-V and glycoprotein IIb-IIIa complexes after endothelial injury or activation.^1^ In normal conditions, VWF has a closed conformation resistant to proteolysis by ADAMTS13, a specific metalloprotease that breaks down uncoiled VWF.^1^ The shear stress of AS caused by blood flowing through a narrow orifice has multiple effects on VWF, uncoiling the VWF multimer and making the VWF multimer sensitive to ADAMTS13 by exposing the bond between Tyr842 and Met843.^1,11-13^ ADAMTS13 cleaves the uncoiled VWF, resulting in acquired VWF deficiency. Further, high shear flow can cause VWF to bind with platelet glycoprotein Ib longer than usual by exposing VWF adhesive surfaces. This process results in small platelet VWF aggregates. These small aggregates are sequestered by natural VWF cleaving protease, resulting in decreased platelet count.^14-16^ The binding of VWF with platelets because of high shear flow and subsequent sequestration explains our patient's low platelet count.

The process of angiodysplasia development from VWF deficiency is complex. Randi et al demonstrated that inhibition of VWF in vitro is associated with increased proliferation, migration, and tube formation related with angiogenesis.^10^ Similar findings were reported from an in vivo experiment with VWF-deficient mice.^17^

The vascular endothelial growth factor (VEGF) family and angiopoietin-1 (Ang-1)/Tie2 system play an important role in angiogenesis, and dysregulation can lead to angiodysplasia.^10^ VEGF plays a predominant role in the initiation and progression of vascular development.^1^ The best characterized protein and receptor are VEGF-A and VEGFR2, respectively.^1^ When VEGF-A binds to VEGFR2 on endothelial cells, it stimulates receptor dimerization and autophosphorylation of intracellular tyrosine kinase residue, causing intracellular signaling cascades that are principally responsible for cell survival, permeability, migration, and proliferation.^10,18,19^ In vivo studies, however, have shown that even though VEGF is known to promote angiogenesis, overexpression of VEGF leads to formation of fragile capillaries and disrupted structures and can lead to angiodysplasia.^10,20^

The Ang-1/Tie2 system is involved in maturation and stability of the newly formed blood vessels.^21^ Ang-1, produced by non–endothelial cells such as pericytes and mural cells, binds to the Tie2 receptor to promote quiescence and stability.^10,21^ However, some experimental models also suggest that Ang-1 is responsible for proliferation of blood vessels.^21^ Proposals that attempt to account for this difference state that the location of the Tie2 receptor protein and its cell surface partners determine whether Ang-1 will act as proliferator or stabilizer.^22^

Ang-1 and Tie2 interaction has also been shown to reduce inflammation.^21^ Ang-1 is a complement to VEGF. While VEGF acts as proliferator, Ang-1 acts as stabilizer.^10^ Their coexpression leads to the growth and stability of blood vessels.

Ang-2 is the antagonistic ligand to the Tie2 receptor. Ang-2 competes with Ang-1 to bind with Tie2, and when bound, promotes vascular destabilization, growth, and inflammation, as opposed to stabilization promoted by Ang-1. To promote angiogenesis, Ang-2 appears to act synergistically with VEGF.^10,23,24^

Increased VEGFR2 phosphorylation has been reported in the microvasculature of VWF-deficient mice.^17^ Therefore, decreased VWF seems to be associated with overgrown, unstable, and fragile vessels characteristic of angiodysplasia.^25^ How VWF modulates VEGFR2 is currently an area of research. One hypothesis is that integrin αvβ3, a VWF ligand, binds to VWF to regulate VEGFR2.^26^ However, the exact signaling that follows this binding is yet to be described.^27^ In vitro studies have shown that VWF-deficient endothelial cells increased the release of Ang-2 from Weibel-Palade bodies, the storage granules of endothelial cells.^27,28^ Increased Ang-2 can lead to unstable angiogenesis.^23,24^

A carbohydrate-binding protein called galectin-3 (Gal-3) plays a role in VWF-dependent angiogenesis. Gal-3 can bind to VWF, VEGFR2, and integrin αvβ3, causing a complex process that has a proangiogenic effect.^29,30^ A Gal-3 inhibitor can reduce angiogenesis. In clinical trials, Gal-3 inhibitors exhibited a good safety profile and may be an additional treatment option for patients with recurrent GI bleed, such as our patient.^27,31^

While the above discussion sheds some light on the process of angiodysplasia, the exact pathophysiological process that results in a Dieulafoy lesion, a specific type of angiodysplasia, is unknown.^32^ Unlike a normal arteriole in the GI mucosa, a Dieulafoy lesion tends to maintain its caliber instead of narrowing as a normal arteriole would. Because the lesion fails to taper in caliber, the arterial branch tends to penetrate the outer layer of the mucosal wall.^7,33^ The vessel is histologically normal. The lesion lacks signs of inflammation or deep ulceration. Arteriosclerosis, aneurysms, elastic tissue abnormalities, or signs of vasculitis are usually not seen.^8,34,35^ Superficial mucosal erosion is considered the primary event leading to arterial rupture.^36,37^ The pulsatility of the large submucosal artery inflicts local microtrauma and ischemia to the mucosa, causing the lesion to thin over time. Initial rupture can occur from minor resistance in the arterial wall and can cause further rupture of the basement membrane and overlying epithelium, leading to localized ischemia that causes erosion and complete rupture.^38-41^^.^

## CONCLUSION

The exact process of how a Dieulafoy lesion, a specific type of angiodysplasia, forms is unknown. This lack of knowledge is noteworthy because of the high mortality associated with Dieulafoy lesions. Because of the increasing incidence of Heyde syndrome among the elderly, documentation of potentially life-threatening complications is important. Our case provides evidence that a Dieulafoy lesion is a rare but potential complication of Heyde syndrome.

## Notes

This article meets the Accreditation Council for Graduate Medical Education and the American Board of Medical Specialties Maintenance of Certification competencies for Patient Care and Medical Knowledge.
